# The diagnostic/prognostic potential and molecular functions of long non-coding RNAs in the exosomes derived from the bile of human cholangiocarcinoma

**DOI:** 10.18632/oncotarget.19547

**Published:** 2017-07-25

**Authors:** Xianxiu Ge, Youli Wang, Junjie Nie, Quanpeng Li, Lingyu Tang, Xueting Deng, Fei Wang, Boming Xu, Xiaochao Wu, Xiuhua Zhang, Qiang You, Lin Miao

**Affiliations:** ^1^ Institute of Digestive Endoscopy and Medical Center for Digestive Diseases, Second Affiliated Hospital of Nanjing Medical University, Nanjing, China; ^2^ Department of Clinical Laboratory, Nanjing First Hospital, Nanjing, China; ^3^ Department of Biotherapy, Second Affiliated Hospital of Nanjing Medical University, Nanjing, China

**Keywords:** cholangiocarcinoma, exosome, long non-coding RNAs, RNA sequencing, functional analysis

## Abstract

Cholangiocarcinoma (CCA) is an aggressive malignancy associated with unfavorable prognosis, and it’s difficult to diagnose and no effective treatments are available. Long non-coding RNAs (lncRNAs) play important roles in tumorigenesis and metastasis. Intact lncRNAs from exosomes have sparked much interest as potential biomarker for the non-invasive analysis of disease. Here, via exosome sequencing on lncRNAs, GO analysis, KEGG pathway and co-expression analysis, receiver operating characteristic curve and survival analyses, we found that, compared with control group, lncRNAs of ENST00000588480.1 and ENST00000517758.1 showed significantly increased expressions in CCA group. Moreover, area under the curve (AUC) was increased to 0.709 when combined the two lncRNAs, they had a sensitivity and specificity of 82.9% and 58.9% respectively. Further, the higher levels of the two lncRNAs showed a significantly increasing trend with the advancement of cancer TNM stages, and prognosticated a poor survival. In addition, KEGG pathway analysis showed that the most significant difference term was “p53 signaling pathway” (KEGG ID: hsa04115, p: 0.001). The altered lncRNAs and their target genes were included to reconstruct a co-expression network. These altered lncRNAs were mainly related to cellular processes, environmental information processing and organismal systems, etc. Collectively, our findings provided the potential roles of lncRNAs of ENST00000588480.1 and ENST00000517758.1 in CCA, and implicated these lncRNAs as potential diagnostic and therapeutic targets for CCA.

## INTRODUCTION

Cholangiocarcinoma (CCA) is an incurable and lethal cancer due to its silent clinical characters, difficulties in early diagnosis, and limited therapeutic approaches. Biopsy and cytology brush biopsy examination is the current gold standard for diagnosis, but it is very difficult to obtain the cells, and the sensitivity is only about 20% [1]. Surgery is an effective means of treatment, but 70% of patients lose the opportunity to surgery because failure of early diagnosis [2]. Therefore, new diagnosis and treatment approaches of CCA are clearly needed.

Tumor-derived exosomes are emerging as a new type of cancer biomarker [3]. Exosomes are 30-100 nm membrane vesicles secreted by a variety of cell types, including tumor cells, it can be generally detected in body fluids such as Serum, urine, saliva, bile, ascites, amniotic fluid, milk, etc [4]. Exosomes are one type of extracellular vesicles (EVs) according to their size and they have been the topic of great interest in recent years in medical research [5]. Exosomes are known to deliver diverse molecules to target cells ranging from mRNAs, non-coding RNAs (ncRNAs) to proteins, which have the diagnostic potential. It is reported that circulating exosomal microRNAs have diagnostic potential for Lung Cancer [6], colon cancer [7], and esophageal squamous cell carcinoma [8]. Combination of serum proteins and microRNAs can increases sensitivity and specificity of pancreatic cancer [9]. Biliary vesicle microRNA-based panel for CCA diagnosis demonstrate a sensitivity of 67% and specificity of 96% [10]. So it is significant to explore and definite the potential functions of the cargos exosomes.

As a subclass of ncRNAs that are present in exosomes, long-ncRNAs (lncRNAs), which are > 200 nucleotides (nt) in length, regulate gene expression at the epigenetic, transcriptional, and posttranscriptional levels, play important roles in diverse biological processes, including cell proliferation, invasion, differentiation, apoptosis, metastasis and immune response [11]. A recent study showed that lncRNAs microarrays of intrahepatic cholangiocarcinoma (ICC) and paired adjacent noncancerous tissues reveal that lncRNAs are significantly changed [12], however, up to date, changes of lncRNAs in exosomes of CCA remain unclear. In our present study, we used RNA sequencing (RNA-seq) to determine lncRNA repertoires of bile exosome of CCA and benign biliary obstruction patients. In order to explore the function of altered lncRNAs, we also measured the gene expression profile. The enriched pathways, biological process, cellular components and molecular functions were explored using bioinformatics. The relationship between the differentially expressed lncRNAs and genes was also analyzed by constructing the co-expression network.

## RESULTS

### Identification of exosomes in human bile samples

**Figure 1:**
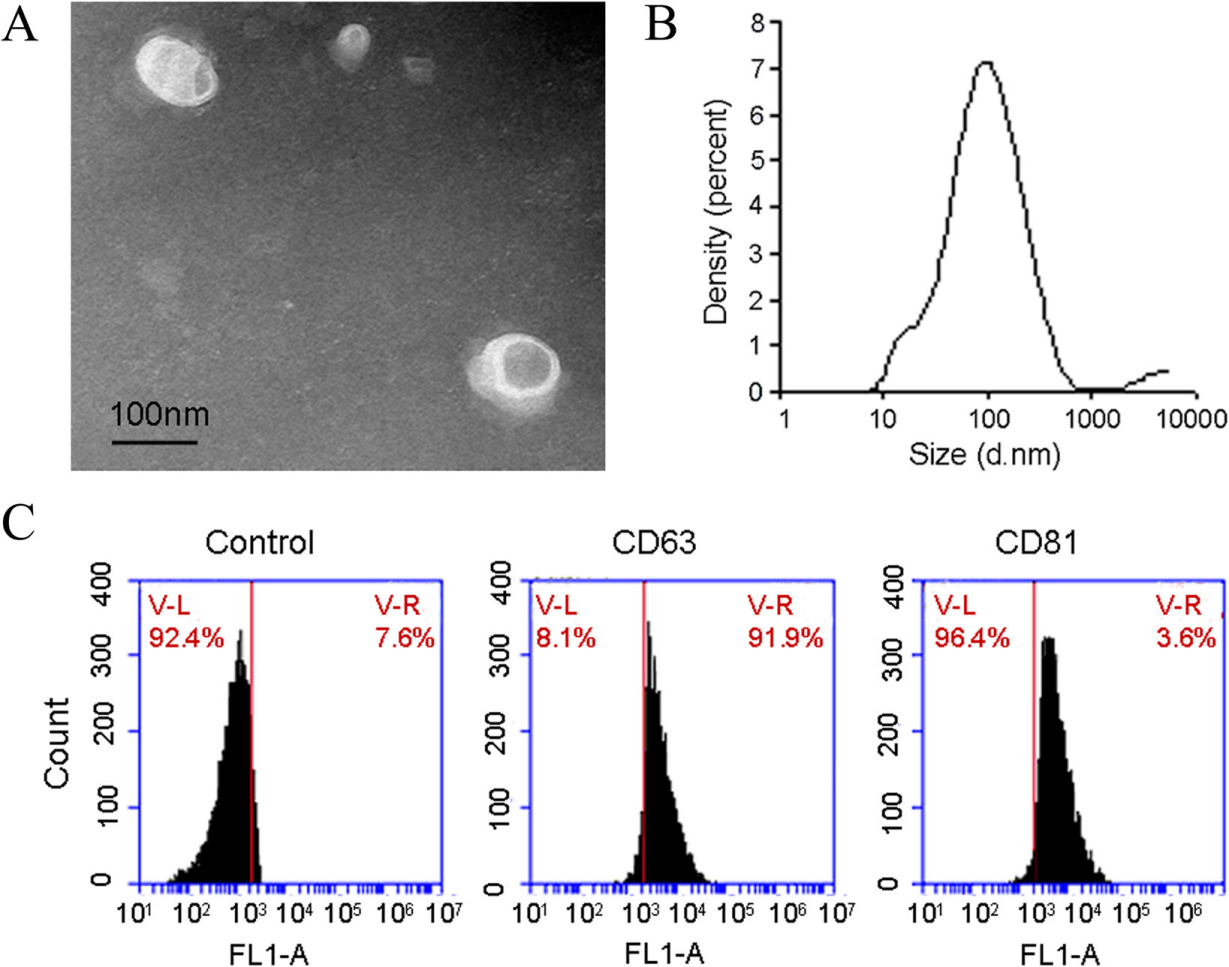
Identification of exosomes in human bile samples **(A)** Typical transmission electron microscopy picture demonstrating the presence of 30-110 nm of spherical structures in human bile. Further characterization demonstrates that these vesicles display exosome characteristics. **(B)** Mode of exosome isolated from human bile was determined to be approximately 72.2 nm (x-axis depicts exosome size and y-axis depicts exosome intensity of each size). **(C)** Presence of typical exosome proteins (CD63 and CD81) in exosome preparations from human bile.

Exosomes isolated from human bile were imaged by using Transmission electron microscope, TEM. We noted the presence of 30-150 nM of vesicles, consistent with previously reported features of exosomes (Figure 1A). Then we employed multiparameter nanoparticle tracking analysis (NTA), and found that the majority of EVs in human bile were between 30 and 150 nM and that the mode of exosome sizes was 72.2 nM (Figure 1B), suggesting that EVs isolated are most likely exosomes. The distribution coefficient was 0.436. To further confirm that these spherical structures are exosomes, we assayed for presence of CD63 and CD81, Flow cytometry analysis positive expression of CD63 was 96.4% and CD81 was 91.9%. (Figure 1C).

### Differentially expressed lncRNAs

**Figure 2:**
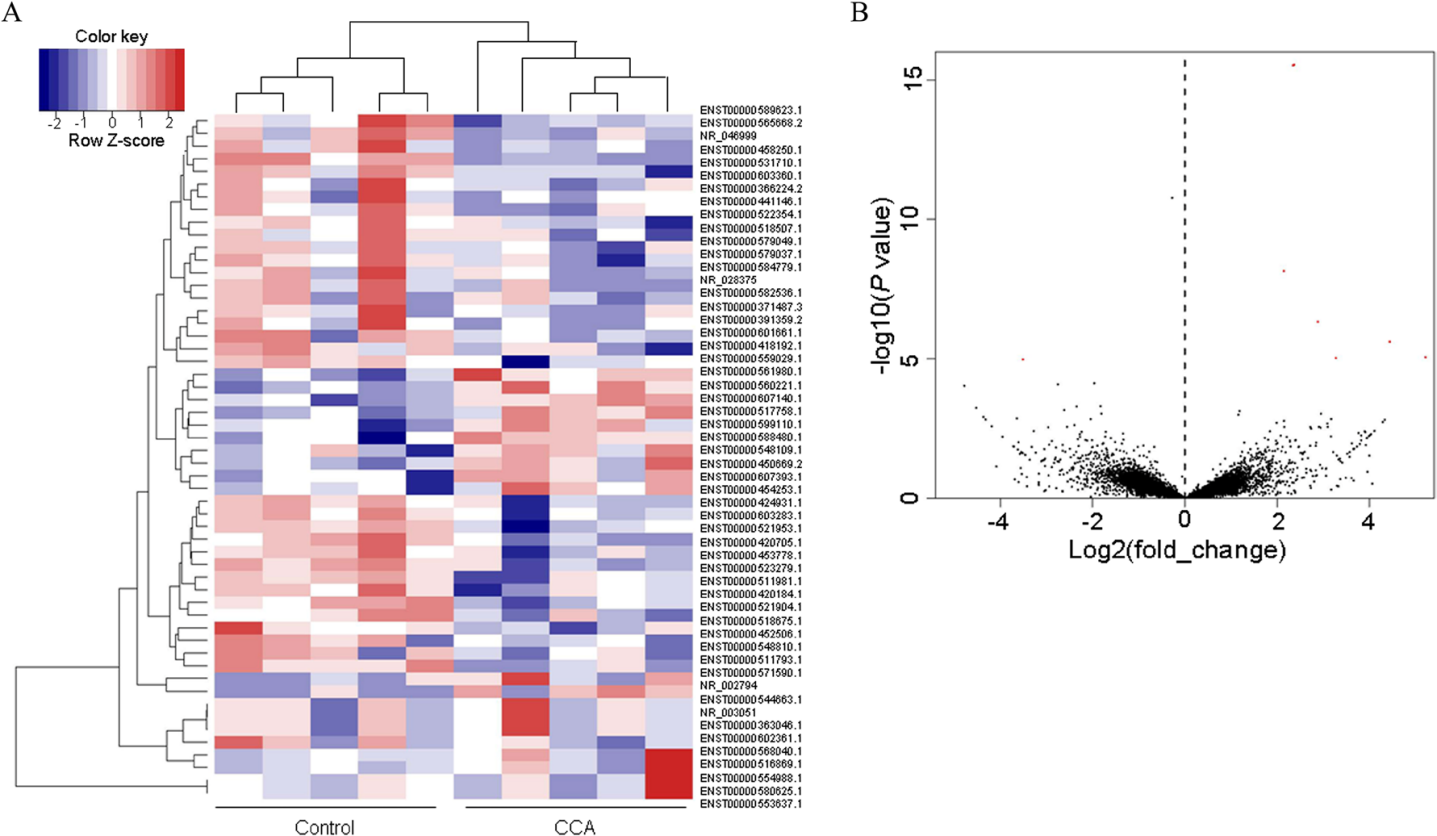
The heat map and volcano plots **(A)** Heat maps showing unsupervised clustering of CCA and controls based on differentially expressed up-regulated lncRNAs (red) and long non-coding genes (blue). **(B)** Volcano plots of all the detected transcripts in both the CCA and biliary obstruction groups. The vertical dotted line delimits up-and down-regulation. Red plots: significant lncRNAs.

A total of 64, 530 transcripts were detected in the current sequencing study, including 30632 transcripts identified as lncRNA. Comparing the mean value of Reads Per Kilobase Million (RPKM) of the CCA group with that of the normal group, 54 transcripts were selected from the 30632 lncRNAs according to the criteria described in the materials and methods section (19 up-regulated and 35 down-regulated). The top 15 up-regulated and 15 down-regulated lncRNAs were listed in Table 1. Their heat map and volcano plots were displayed in Figures 2A and 2B. Briefly, data normalized by Z score transformation were used in the calculation of significant changes in expression in the Control and CCA group, and the transformed data were used to construct the heatmap. The red signal referred to high relative expression and the blue signal referred to low relative expression.

**Table 1:** Different expression of lncRNAs

lncRNA	log2^(Fold change)^	*p*-Value	lncRNA	log2^(Fold change)^	*p*-Value
Top 15 up-regulated lncRNAs			Top 15 down-regulated lncRNAs		
ENST00000544663.1	3.571	0.033	ENST00000571590.1	-3.054	ENST00000571590.1
NR_002794	3.220	0.023	ENST00000548810.1	-2.316	ENST00000548810.1
ENST00000516869.1	2.350	0.000	ENST00000584779.1	-2.272	ENST00000584779.1
ENST00000554988.1	2.152	0.000	ENST00000518675.1	-2.257	ENST00000518675.1
ENST00000607140.1	2.080	0.006	ENST00000521904.1	-2.221	ENST00000521904.1
ENST00000454253.1	1.911	0.031	ENST00000523279.1	-2.145	ENST00000523279.1
ENST00000517758.1	1.900	0.021	ENST00000511793.1	-2.086	ENST00000511793.1
ENST00000599110.1	1.807	0.049	ENST00000531710.1	-2.027	ENST00000531710.1
ENST00000580625.1	1.782	0.000	ENST00000579049.1	-2.007	ENST00000579049.1
ENST00000553637.1	1.782	0.000	ENST00000441146.1	-2.004	ENST00000441146.1
ENST00000607393.1	1.677	0.028	ENST00000452506.1	-1.970	ENST00000452506.1
ENST00000588480.1	1.668	0.026	ENST00000458250.1	-1.955	ENST00000458250.1
ENST00000450669.2	1.588	0.017	ENST00000589623.1	-1.921	ENST00000589623.1
ENST00000560221.1	1.482	0.042	ENST00000565668.2	-1.898	ENST00000565668.2
ENST00000561980.1	1.424	0.046	ENST00000579037.1	-1.804	ENST00000579037.1

### Gene ontology (GO) and **kyoto** encyclopedia of genes and genomes (KEGG) pathway enrichment analysis of the differentially expressed genes

**Figure 3:**
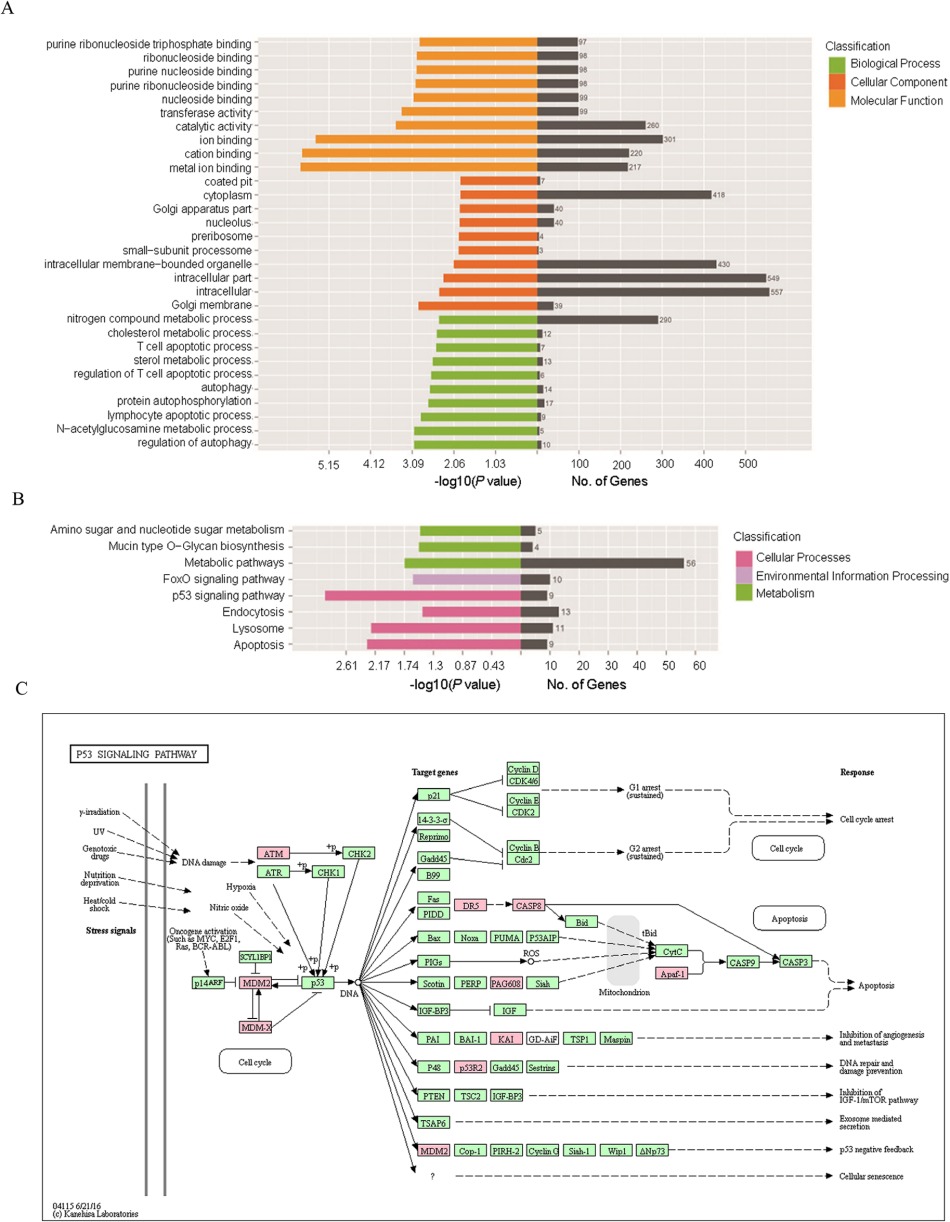
GO and KEGG pathway analysis of the differentially expressed genes **(A)** GO enrichment for the cluster analysis of the pathways related to biological processes, cellular components and molecular functions. Horizontal axis denoted the respective -Log10 (p-value) of different pathways and the vertical axis represented the pathway name. Numbers of genes in each cluster are shown in the right black histogra. **(B)** KEGG enrichment for the Cluster analysis of the pathways related to cellular processes, environmental information processing, genetic information processing, human diseases as well as organismal systems. Horizontal axis denoted the respective -Log10 (*p*-value) of different pathways and the vertical axis represented the pathway name. Numbers of genes in each cluster are shown in the right black histogra. **(C)** Detailed network of “P53 signaling pathway”. Every node represented the key enzyme or transporter in this pathway. Nodes marked in red were associated with significantly up-regulated genes in the current sequencing data, and the green nodes denoted genes with no significant change.

A total of 4002 terms in the GO-biological process were involved in current sequencing data. The top 10 biological process terms with the highest *p*-value were listed in Table 2, and the most significant process was related to “Regulation of autophagy”. The top 10 cellular components with the highest *p*-value were shown in Table 3, and the most significant process was related to “Golgi membrane”. The top 10 molecular functions terms were displayed in Table 4, and the most significant one was “Metal ion binding”. GO enrichment for the Cluster analysis of the pathways was shown in Figure 3A.

**Table 2:** Top 10 GO biological processes

GOBPID	Sample number	Background number	*P* value	Term
GO:0010506	10	71	2.085E-04	regulation of autophagy
GO:0006044	5	16	2.207E-04	N-acetylglucosamine metabolic process
GO:0070227	9	62	2.933E-04	lymphocyte apoptotic process
GO:0046777	17	188	4.304E-04	protein autophosphorylation
GO:0006914	14	141	5.827E-04	autophagy
GO:0070232	6	31	7.679E-04	regulation of T cell apoptotic process
GO:0016125	13	128	1.523E-03	sterol metabolic process
GO:0070231	7	45	1.701E-03	T cell apoptotic process
GO:0008203	12	116	1.876E-03	cholesterol metabolic process
GO:0006807	290	6306	1.933E-03	nitrogen compound metabolic process

“GOBPID”: the ID of biological process in GO database; “Sample number”: the number of candidate genes; “Background number”: the number of background genes in the pathway; “*P* value”: significance level; “Term”: pathway function.

**Table 3:** Top 10 GO cellular components

GOCCID	Sample number	Background number	*P* value	Term
GO:0000139	39	574	0.001	Golgi membrane
GO:0005622	557	12706	0.004	intracellular
GO:0044424	549	12559	0.005	intracellular part
GO:0043231	430	9789	0.009	intracellular membrane-bounded organelle
GO:0032040	3	10	0.011	small-subunit processome
GO:0030684	4	20	0.011	preribosome
GO:0005730	40	689	0.012	nucleolus
GO:0044431	40	690	0.012	Golgi apparatus part
GO:0005737	418	9569	0.013	cytoplasm
GO:0005905	7	60	0.013	coated pit

“GOCCID”: the ID of cellular component in GO database.

**Table 4:** Top 10 GO molecular functions

GOMFID	Sample number	Background number	*P* value	Term
GO:0046872	217	3944	1.39E-06	metal ion binding
GO:0043169	220	4015	1.54E-06	cation binding
GO:0043167	301	5849	3.32E-06	ion binding
GO:0003824	260	5326	3.18E-04	catalytic activity
GO:0016740	99	1762	4.43E-04	transferase activity
GO:0001882	99	1802	8.74E-04	nucleoside binding
GO:0032550	98	1788	9.9E-04	purine ribonucleoside binding
GO:0001883	98	1791	1.039E-03	purine nucleoside binding
GO:0032549	98	1792	1.056E-03	ribonucleoside binding
GO:0035639	97	1780	1.233E-03	purine ribonucleoside triphosphate binding

“GOMFID”: the ID of molecular function in GO database.

KEGG pathway analysis indicated that 240 pathways were involved in the current sequencing data, in which many pathways were related to cellular processes, environmental information processing, genetic information processing, human diseases as well as organismal systems (Figure 3B). The most enriched top ten pathway terms with the lowest *p*-value were p53 signaling pathway, apoptosis, lysosome, metabolic pathways, FOXO signaling pathway, mucin type O-Glycan biosynthesis, amino sugar and nucleotide sugar metabolism, endocytosis, sulfur metabolism, tryptophan metabolism. Further details were shown in Table 5. The most significant difference term was “p53 signaling pathway” (KEGG ID: hsa04115, p: 0.001), which involved 9 target genes and the network (Figure 3C).

**Table 5:** Top 10 KEGG pathways

Term	ID	Sample number	Background number	*P*-value
p53 signaling pathway	hsa04115	9	68	0.001
Apoptosis	hsa04210	9	86	0.005
Lysosome	hsa04142	11	122	0.006
Metabolic pathways	hsa01100	56	1181	0.018
FoxO signaling pathway	hsa04068	10	133	0.025
Mucin type O-Glycan biosynthesis	hsa00512	4	31	0.030
Amino sugar and nucleotide sugar metabolism	hsa00520	5	47	0.031
Endocytosis	hsa04144	13	203	0.034
Sulfur metabolism	hsa00920	2	10	0.061
Tryptophan metabolism	hsa00380	4	40	0.062

“Term”: pathway function; “ID”: pathway ID; “Sample number”: the number of candidate genes; “Background number”: the number of background genes in the pathway; “*P* value”: significance level.

### Construction of lncRNAs-proteins co-expression network

**Figure 4:**
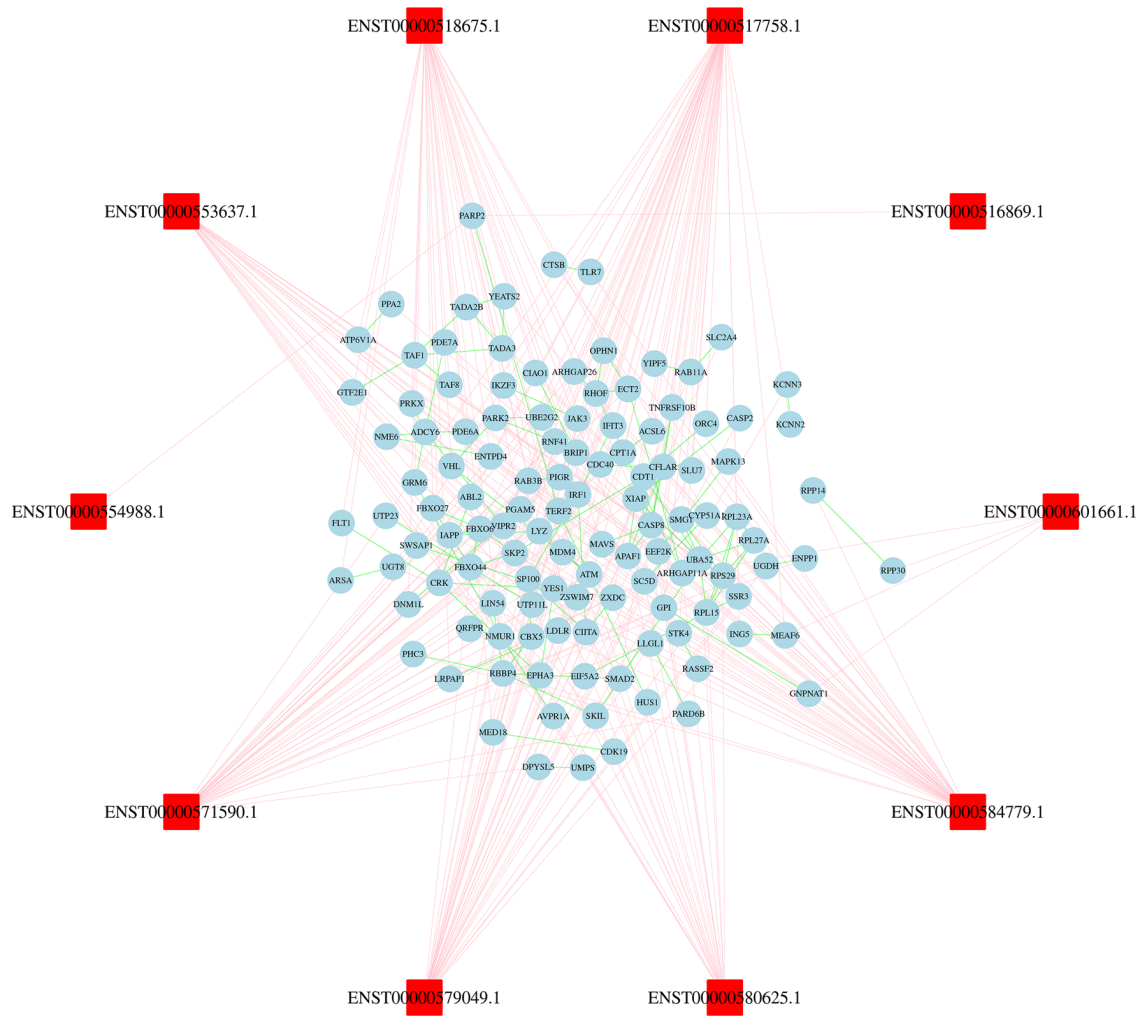
Construction of lncRNAs-proteins co-expression network The blue oval represented gene, and the red rectangle represented lncRNA. The pink lines link gene to lncRNA, green lines link gene to gene.

We then evaluated the co-expression network of lncRNAs and their target genes based on the criterion of combined score > 0.9. In this co-expression network, the co-expression of lncRNA-gene and gene-gene was showed by lines of different colors (Figure 4). The lncRNA-gene pair with the highest positive correlation coefficient (0.993) was CYP51A1 and SC5D. These results indicated that one lncRNA closely correlated with several genes, and vice versa. The network can indicate functional relatedness or regulatory relationships [13].

### Validation of diagnostic and prognostic value of lncRNAs in CCA

**Figure 5:**
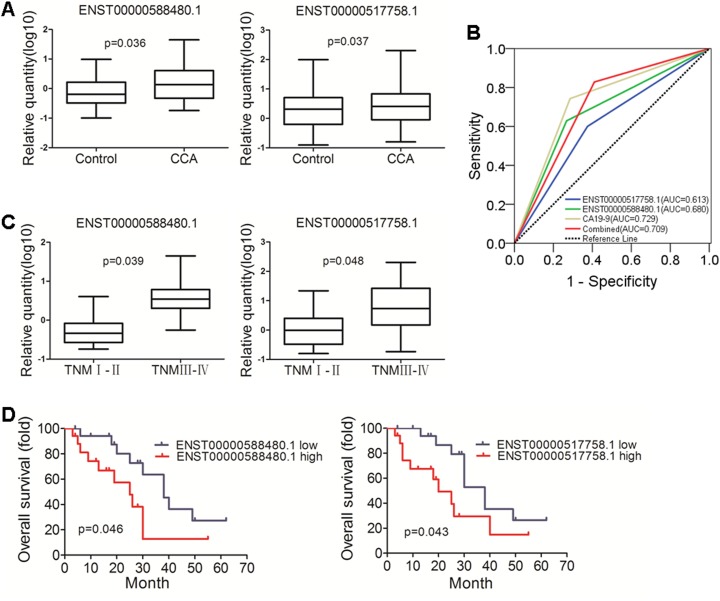
Validation of diagnostic and prognosis value of lncRNAs in CCA **(A)** Different expression of ENST00000588480.1 and ENST00000517758.1, the two lncRNAs were up-regulated in CCA group (*p* = 0.036 and 0.037). **(B)** ROC curve of lncRNAs and CA19-9. The analysis showed the AUC of CA19-9 was 0.729 (95% CI, 0.620 - 0.837, it had a sensitivity and specificity of 74.3% and 71.4%; AUC of ENST00000588480.1 was 0.680 (95% CI, 0.565 - 0.796), it had a sensitivity and specificity of 62.9% and 73.2%; When ENST00000588480.1 and ENST00000517758.1 were combined, They got greater sensitivity (82.9%) (AUC: 0.709; 95% CI, 0.601-0.817). **(C)** The levels of ENST00000588480.1 and ENST00000517758.1 showed a significant increacing trend with the advancement of cancer TNM stages. (*p* = 0.039 and 0.048). **(D)** Kaplan-Meier survival overall survival (OS) curves for ENST00000588480.1 and ENST00000517758.1, The ENST00000588480.1 demonstrated statistically significant median overall survival differences (38 vs 25 months, *p* = 0.046) compared to ENST00000517758.1 (38 vs 20 months, *p* = 0.043).

We detected exosomes of differentially expressed lncRNAs between bile specimens of CCA and the control patients. Significant different expression lncRNAs: ENST00000588480.1 and ENST00000517758.1 were selected for further detection using real-time RT-PCR in bile samples of CCA (n=35) and the control patients (n=56) (Figure 5A). These results were higher than those shown in the expression RNA-Seq data sets. To confirm the performance of the two lncRNAs in diagnosing CCA, the receiver operating characteristic curves (ROC) were applied and the AUC of each curve was calculated (Figure 5B). The result indicates that the diagnosis of CCA is more efficient in the combination of the two lncRNAs than any single ones. Furthermore, the levels of ENST00000588480.1 and ENST00000517758.1 showed a significantly increasing trend with the advancement of cancer TNM stages, Figure 5C). The relationships of the two lncRNAs with prognosis status of CCA patients were further examined by Kaplan-Meier curve analysis and log-rank test (Figure 5D).

### LncRNA signature-associated signalling pathways

**Figure 6:**
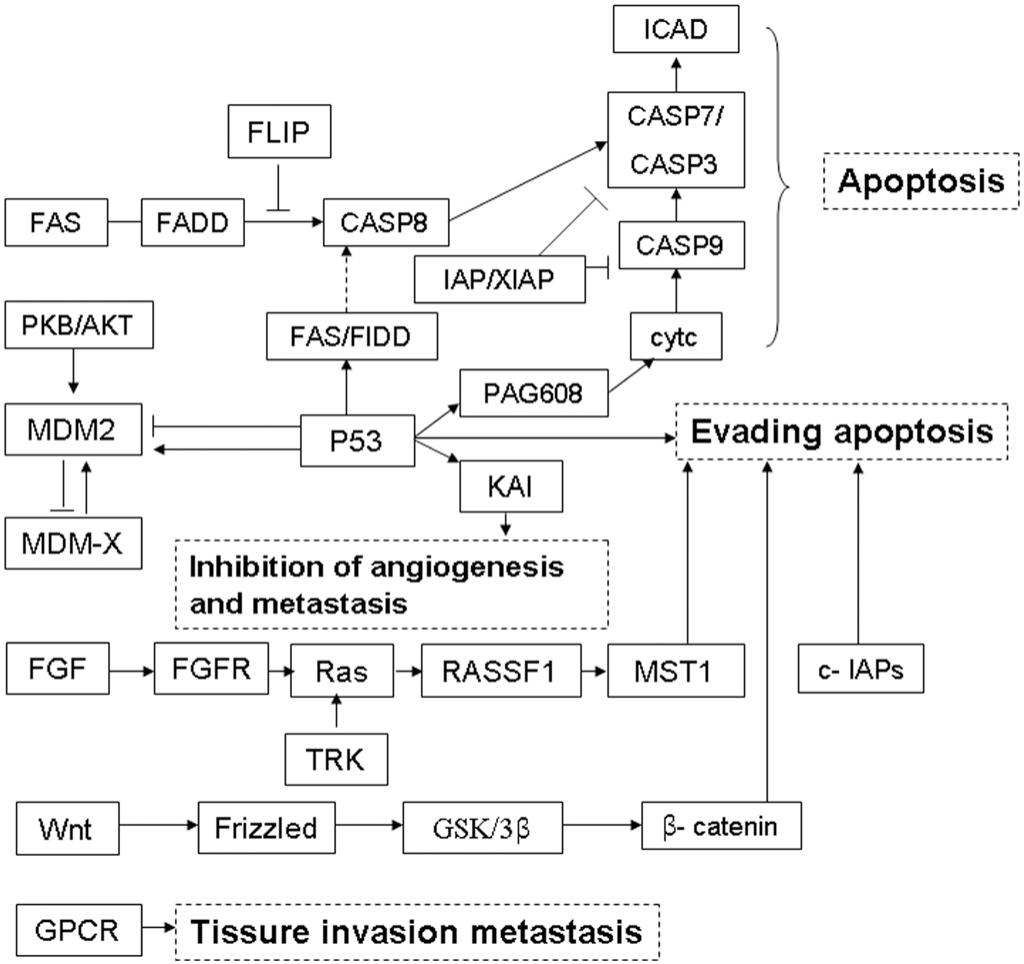
LncRNA signature-associated signalling pathways A schematic diagram showed the major biological functions closely associated to were apoptosis, angiogenesis, invasion and metastasis etc.

A gene KEGG pathway and Go analysis was performed according to the meterial and methods section. 479 target genes were concerned to form a gene list, while 17 of them were enriched in 3 pathways: “P53 signaling pathway”, “apoptosis” and ENST00000588480.1 and ENST00000517758.1. A chart was drown to explore the associated genes and pathways of the three lncRNAs for further mechanism study (Figure 6). The major biological functions closely associated were apoptosis, angiogenesis, invasion and metastasis etc.

## DISCUSSION

Exosomes, endosome-derived membrane microvesicles, contain specific RNA transcripts that are thought to be involved in cell-cell communication [14]. Exosomes in body fluids are relatively stable because of the protection of the lipid bilayer, the cargos they contain have great potential as disease biomarkers [15, 16]. It was reported that plasma lncRNA protected by exosomes as a potential stable biomarker for gastriccancer [17]. In urine samples, exosomal lncRNA-p21 levels may help to distinguish prostate cancer from benign disease [18]. Exosomal lncRNAs may also be potential biomarkers for predicting diseases [18, 19]. Besides, they can activate or regulate cell activities such as protein expression, cell proliferation and differentiation or antiviral responses [20]. Therefore, in the current study, we isolated exosomes from human bile samples for RNA-seq. Differently expressed lncRNAs were identified using qPCR method. Ultimately, three lncRNAs closely associated with CCA were selected and both diagnostic and prognostic values were examined. Combined group (ENST00000588480.1 and ENST00000517758.1) got greater sensitivity than carbohydrate antigen 19-9 (CA19-9) (82.9% vs 74.3%). We proposed that the diagnostic ability of the two lncRNAs is robust because of independent cohort of 35 CCA patients. To our knowledge, this is the first study to give a comprehensive overview of the lncRNA transcriptome changes in CCA and biliary obstruction of bile exosomes. They showed a significant increasing trend with the advancement of cancer TNM stages. Regarding prognosis, Kaplan-Meier curve analysis the higher expression group were negative correlate with the survival time. Therefore, lncRNAs can be used as a predictor to monitor the CCA.

LncRNAs were detected in exosomes of body fluid, and they were enriched in exosomes than cells [16], and of high tissue specificity [21]. The function of diagnosis potential were sufficiently reported in cancers [22–25]. Except for that, numerous studies have shown that lncRNAs have the function of regulate gene expression at different levels including chromatin modifications, transcription, splicing, translation, posttranscriptional regulation, processing of small RNAs, etc [26]. It was reported that it play active roles in various biological behaviors of tumor cells such as migration, genomic stability, immune modulation and drug resistance. For instance, Lung cancer exosomes initiate lncRNA changes in mesenchymal stem cells, lncRNAs may be involved in exosome-mediated crosstalk between MSCs and tumor cells [27]. exosomes containing H19 lncRNA OF cancer stem-cell-like (CSC) liver cancer cells can influence their tumor microenvironment by promoting angiogenesis [28]. Extracellular Vesicle lincRNA-VLDLR was relevant to the chemoresistance in hepatocellular carcinoma [29]. So here, we further determined the potential functions mediated by the changed lncRNAs in CCA bile exosomes.

GO and KEEG pathway analyses showed that many important functional pathways were closely related to the altered genes. Further analysis showed that the pathways involved were mainly related to cellular processes, environmental information processing as well as genetic information processing. These results emphasized the important role of micro-environmental information processing on the cellular function. Constant with the previous studies supported that lncRNAs in the tumor inflammation [30] or hypoxic microenvironment [31] may resulting in an imbalance of internal environment, which enhancing the progression of cancer progression. Although part of the variability in lncRNA repertoire size may be biologically meaningful, much is likely to be explained by unequal sequencing depth and by variable genome sequence and assembly quality.

Because the two lncRNAs were differently expressed not only between CCA and contol bile samples, but also between well- and poor prognosis patients, we speculated that these lncRNAs may play critical roles in both tumourigenesis and the progression of CCA, and that both of the two lncRNAs may be potential therapeutic targets in CCA. To preliminarily reveal the potential functions of the lncRNAs in CCA, the mRNAs co-expressed with each of the three lncRNAs were used to conduct KEGG and GO analysis in KOBAS, the website is http://kobas.cbi.pku.edu.cn/index.php. ENST00000588480.1 enriches the Hedgehog signaling pathway, and may have the function of ‘lipid biosynthetic process’, ‘Wnt signaling pathway’, “binding of glycoprotein, ribosomal protein and phosphoprotein”, “peptide kinase and phosphotransferase activity”. ENST00000517758.1 enriches “p53 signaling pathway”, “Pathways in cancer” and “Endocytosis” etc. and may have the function of “Metal ion binding”, “regulation of leukocyte and T cell apoptotic process”, “cholesterol metabolism” and “transmembrane transporter activity”. These informations may improve the current understanding of the close association between the two lncRNAs and CCA, but the underlying mechanism has not been deciphered. Additional prospective and multicentre studies with a considerable pool of patients are needed to determine whether this signature is applicable for CCA.

## MATERIALS AND METHODS

### Patient samples and bile exosome isolation

Bile samples of CCA (n = 35, Supplementary Table 1) and biliary obstruction patients (n = 56) were obtained by endoscopic retrograde cholangiopancreatography (ERCP) or surgery from the second affiliated hospital of Nanjing Medical University, aspiration of bile was performed after cannulation of the biliary tree before injection of contrast. All work presented here was approved by second affiliated hospital of Nanjing Medical University Review Board. All patients provided written informed consent. We prepared about 4ml of bile samples for use, They were centrifuged at 500g for 10 minutes at 4°C to pellet cells and debris. The supernatant was then centrifuged at 16500g for 20 minutes at 4°C to further remove cellular debris and then filtered through a 200-nm filter. Then Pipet the filtered supernatant to ultracentrifuge tubes and add PBS so that the tubes are more than 2/3 full, ultracentrifugate at 120000 g for 70 min at 4°C, then we can get small yellow pellets at the bottom of the ultracentrifuge tube. Exosomes were utilized for immediate RNA sequencing, RNA extraction or resuspended in phosphate buffered saline and stored in -80°C.

### Functional assays

Please see the SUPPLEMENTARY MATERIALS AND METHODS section.

### Statistical analysis

All the biological specimens were analysed retrospectively. Data were presented as the min to max. Statistical analyses were performed using Graph pad 6.0 and/or IBM SPSS Statistics 17.0 software, differences were considered statistically significant when the p < 0.05. The difference between CCA and control group was analyzed using a Mann-Whitney test. Then CCA patients were divided into high and low groups according to the median of the lncRNA expression, survival curves were estimated using the Kaplan-Meier method, and differences in survival distributions were evaluated by the log-rank test.

## SUPPLEMENTARY MATERIALS FIGURES AND TABLES



## Abbreviations

(CCA): cholangiocarcinoma
(GO): gene ontology
(KEGG): kyoto encyclopedia of genes and genomes
(lncRNAs): long non-coding RNAs
(ROC): receiver operating characteristic curve
(nt): nucleotides
(RPKM): Reads Per Kilobase Million
(AUC): area under the curve

## References

[1] Blechacz B, Komuta M, Roskams T, Gores GJ (2011). Clinical diagnosis and staging of cholangiocarcinoma. Nat Rev Gastroenterol Hepatol.

[2] Marsh Rde W, Alonzo M, Bajaj S, Baker M, Elton E, Farrell TA, Gore RM, Hall C, Nowak J, Roy H, Shaikh A, Talamonti MS (2012). Comprehensive review of the diagnosis and treatment of biliary tract cancer 2012. Part I: diagnosis-clinical staging and pathology. J Surg Oncol.

[3] Whiteside TL (2015). The potential of tumor-derived exosomes for noninvasive cancer monitoring. Expert Rev Mol Diagn.

[4] Raimondo F, Morosi L, Chinello C, Magni F, Pitto M (2011). Advances in membranous vesicle and exosome proteomics improving biological understanding and biomarker discovery. Proteomics.

[5] Lemoinne S, Thabut D, Housset C, Moreau R, Valla D, Boulanger CM, Rautou PE (2014). The emerging roles of microvesicles in liver diseases. Nat Rev Gastroenterol Hepatol.

[6] Rabinowits G, Gercel-Taylor C, Day JM, Taylor DD, Kloecker GH (2009). Exosomal microRNA: a diagnostic marker for lung cancer. Clin Lung Cancer.

[7] Ogata-Kawata H, Izumiya M, Kurioka D, Honma Y, Yamada Y, Furuta K, Gunji T, Ohta H, Okamoto H, Sonoda H, Watanabe M, Nakagama H, Yokota J (2014). Circulating exosomal microRNAs as biomarkers of colon cancer. PLoS One.

[8] Tanaka Y, Kamohara H, Kinoshita K, Kurashige J, Ishimoto T, Iwatsuki M, Watanabe M, Baba H (2013). Clinical impact of serum exosomal microRNA-21 as a clinical biomarker in human esophageal squamous cell carcinoma. Cancer.

[9] Madhavan B, Yue SJ, Galli U, Rana S, Gross W, Muller M, Giese NA, Kalthoff H, Becker T, Buchler MW, Zoller M (2015). Combined evaluation of a panel of protein and miRNA serum-exosome biomarkers for pancreatic cancer diagnosis increases sensitivity and specificity. Int J Cancer.

[10] Li L, Masica D, Ishida M, Tomuleasa C, Umegaki S, Kalloo AN, Georgiades C, Singh VK, Khashab M, Amateau S, Li Z, Okolo P, Lennon AM (2014). Human bile contains microRNA-laden extracellular vesicles that can be used for cholangiocarcinoma diagnosis. Hepatology.

[11] Azmi AS, Bao B, Sarkar FH (2013). Exosomes in cancer development, metastasis, and drug resistance: a comprehensive review. Cancer Metastasis Rev.

[12] Wang J, Xie H, Ling Q, Lu D, Lv Z, Zhuang R, Liu Z, Wei X, Zhou L, Xu X, Zheng S (2016). Coding-noncoding gene expression in intrahepatic cholangiocarcinoma. Transl Res.

[13] Stuart JM, Segal E, Koller D, Kim SK (2003). A gene-coexpression network for global discovery of conserved genetic modules. Science.

[14] Milane L, Singh A, Mattheolabakis G, Suresh M, Amiji MM (2015). Exosome mediated communication within the tumor microenvironment. J Control Release.

[15] Malik ZA, Kott KS, Poe AJ, Kuo T, Chen L, Ferrara KW, Knowlton AA (2013). Cardiac myocyte exosomes: stability, HSP60, and proteomics. Am J Physiol Heart Circ Physiol.

[16] Gezer U, Ozgur E, Cetinkaya M, Isin M, Dalay N (2014). Long non-coding RNAs with low expression levels in cells are enriched in secreted exosomes. Cell Biol Int.

[17] Li Q, Shao YF, Zhang XJ, Zheng T, Miao M, Qin LJ, Wang BJ, Ye GL, Xiao BX, Guo JM (2015). Plasma long noncoding RNA protected by exosomes as a potential stable biomarker for gastric cancer. Tumor Biol.

[18] Isin M, Uysaler E, Ozgur E, Koseoglu H, Sanli O, Yucel OB, Gezer U, Dalay N (2015). Exosomal lncRNA-p21 levels may help to distinguish prostate cancer from benign disease. Front Genet.

[19] Tang Q, Ni Z, Cheng Z, Xu J, Yu H, Yin P (2015). Three circulating long non-coding RNAs act as biomarkers for predicting NSCLC. Cell Physiol Biochem.

[20] Sato K, Meng FY, Glaser S, Alpini G (2016). Exosomes in liver pathology. J Hepatol.

[21] Ponting CP, Oliver PL, Reik W (2009). Evolution and functions of long noncoding RNAs. Cell.

[22] Fan ZY, Liu W, Yan C, Zhu ZL, Xu W, Li JF, Su L, Li C, Zhu ZG, Liu B, Yan M (2016). Identification of a five-lncRNA signature for the diagnosis and prognosis of gastric cancer. Tumour Biol.

[23] Mouraviev V, Lee B, Patel V, Albala D, Johansen TE, Partin A, Ross A, Perera RJ (2016). Clinical prospects of long noncoding RNAs as novel biomarkers and therapeutic targets in prostate cancer. Prostate Cancer Prostatic Dis.

[24] Xu B, Shao Q, Xie K, Zhang Y, Dong T, Xia Y, Tang W (2016). The long non-coding RNA ENST00000537266 and ENST00000426615 influence papillary thyroid cancer cell proliferation and motility. Cell Physiol Biochem.

[25] Mohankumar S, Patel T (2016). Extracellular vesicle long noncoding RNA as potential biomarkers of liver cancer. Brief Funct Genomics.

[26] Gibb EA, Vucic EA, Enfield KS, Stewart GL, Lonergan KM, Kennett JY, Becker-Santos DD, MacAulay CE, Lam S, Brown CJ, Lam WL (2011). Human cancer long non-coding RNA transcriptomes. PLoS One.

[27] Wang S, Li X, Zhu R, Han Q, Zhao RC (2016). Lung cancer exosomes initiate global long non-coding RNA changes in mesenchymal stem cells. Int J Oncol.

[28] Conigliaro A, Costa V, Lo Dico A, Saieva L, Buccheri S, Dieli F, Manno M, Raccosta S, Mancone C, Tripodi M, De Leo G, Alessandro R (2015). CD90+liver cancer cells modulate endothelial cell phenotype through the release of exosomes containing H19 lncRNA. Mol Cancer.

[29] Takahashi K, Yan IK, Wood J, Haga H, Patel T (2014). Involvement of extracellular vesicle long noncoding RNA (linc-VLDLR) in tumor cell responses to chemotherapy. Mol Cancer Res.

[30] Wang L, Wu F, Song Y, Li X, Wu Q, Duan Y, Jin Z (2016). Long noncoding RNA related to periodontitis interacts with miR-182 to upregulate osteogenic differentiation in periodontal mesenchymal stem cells of periodontitis patients. Cell Death Dis.

[31] Mineo M, Ricklefs F, Rooj AK, Lyons SM, Ivanov P, Ansari KI, Nakano I, Chiocca EA, Godlewski J, Bronisz A (2016). The long non-coding RNA HIF1A-AS2 facilitates the maintenance of mesenchymal glioblastoma stem-like cells in hypoxic niches. Cell Rep.

