# Supplementary material for: The diagnostic/prognostic potential and molecular functions of long non-coding RNAs in the exosomes derived from the bile of human cholangiocarcinoma
Source: Oncotarget. 2017 Jul 25;8(41):69995–70005. doi: 10.18632/oncotarget.19547 (PMC5642533; doi:10.18632/oncotarget.19547)
Supplement: Supplementary file 1 [file oncotarget-08-69995-s001.pdf]

The diagnostic/prognostic potential and molecular functions of long non-coding RNAs in the exosomes derived from the bile of human cholangiocarcinoma

SUPPLEMENTARY MATERIALS

Exosome identification

To observe the size and shape of exosomes, transmission electron microscopy (TEM) and multi-parameter nanoparticle optical analysis were performed. Exosome-specific membrane proteins such as Alix, TSG101, CD81, CD82, CD63, CD9, CD24, EpCAM, Hsc70 are commonly used. They can be ditected by flow cytometry analysis and western blotting. In our study, we performed Flow cytometry analysis for CD63 and CD81.

TEM

Resuspend extracellular vesicles in 100 µl of PBS. Adsorb 20 µl to 400 mesh carbon-coated Parlodion copper grids for 2 min and allow to dry at RT. Fix the exosome in 1% (v/v) glutaraldehyde (EM-grade) for 5 min at RT by placing drops carefully on the dried preparation. Wash the grids twice with water for 5 min and then contrast stain EVs with 1% phosphotungstic acid (PTA) for 30 sec. Acquire images with an electron microscope with an acceleration voltage of 80 kilovolts starting at magnifications of 20,000X and increasing to 100,000X when determining the size of the particles. Acquire the images at 200 kV with magnifications of Multi-parameter nanoparticle optical analysis. Resuspend the isolated exosomes in 1ml aseptic cold PBS. Slowly poured into a disposable clean sample pool, avoiding bubbles, then sealed the sample pool with a lid. Visualize the exosomes by ZETASIZER Nano series-Nano-ZS (Malvern, U.K.) according to manufacturer's protocol.

Flow cytometry analysis

Resuspend the isolated exosomes with 200µL aseptic cold PBS, then incubated with 20µL CD63-antibody-FITC (BD Biosciences, CA) and CD81-antibody-FITC (BD Biosciences, CA). No antibody labeled as the control, fluorescence intensity of coal maceral were conducted using the Accuri C6 flow cytomenter (BD Biosciences, CA).

Mapping of sequencing reads

All the reads were selected and the clean data were mapped to chromosomes, and the samples from both benign biliary obstruction and CCA groups exhibited very similar chromosome coverage and DNA distribution. In view of the similarity between several sequencing data, we presented the representative data from both benign biliary obstruction and CCA groups. In the sample representing the control group, the total detected reads were 82, 121, 884 (100%), total mapped reads were 72, 186, 375 (87.9%), multiple mapped reads were 2, 409, 464 (2.93%), uniquely mapped reads were 69, 776, 911 (84.97%), reads mapped in proper pairs were 36, 568, 116 (44.53%), reads mapped to “ + ” strand were 35, 618, 259 (43.37%), and reads mapped to “ - ” strand were 36, 049, 876 (43.9%). All the reads were aligned to human chromosomes (chr1-22, X and Y) and centered on several different regions: exons (5.82%), introns (39.04%), and intergenic region (55.14%) (Supplementary Figures 1A and 1B). In the sample representing the CCA group, all the sequencing reads were aligned to human chromosome (chr1-22, X and Y) and the total detected reads were 79, 337, 264 (100%), total mapped reads were 64, 785, 827 (81.66%), multiple mapped reads were 2, 320, 751 (2.93%), uniquely mapped reads were 62, 465, 076 (78.73%), reads mapped in proper pairs were 33, 506, 263 (42.23%), reads mapped to “ + ” strand were 31, 279, 564 (39.43%), and the reads mapped to “ - ” strand were 32, 391, 395 (40.83%). All the detected reads were also mapped to different regions: exonic region (6.34%), intronic region (39.07%), and intergenic region (54.59%) (Supplementary Figures 1C and 1D). The length distribution of exosomal lncRNAs are showed in Supplementary Figure 2.

Real-time reverse transcription-PCR

We used the bile RNA concentration as a quantitative standard. Total RNA was isolated from cells using the Trizol Total RNA Isolation kit (Invitrogen, Carlsbad, CA) according to the manufacturer’s protocol. RNA was eluted with RNasefree water. Reverse transcription (RT)-PCR was performed using the Transcriptor First Strand cDNA Synthesis kit (Roche Molecular Biochemicals, Indianapolis, IN) according to the manufacturer’s protocol. Then the mRNAs were amplified by qRT-PCR with SYBR green (Invitrogen) under the following conditions: 95 °C for 30 sec; 40 cycles of 95 °C for 5 sec; 60 °C for 30 sec; and melting at 95 °C for 5 sec, followed by 60 °C for 1 min, and cooling at 50 °C for 30sec. The mRNAs expression was normalized to that of PLA (details of chosing method was described in the next paragraph).

Fold changes in expression of each gene were calculated by a comparative threshold cycle (Ct) method using the formula $2^{-(\Delta\Delta Ct)}$. The primers used in this study were listed in the Supplementary Table 2.

Reference gene identification

To systematically select reference genes which are applicable in exosome RNA of bile (25 paired CCA and biliary stricture samples), 13 common reference candidates were chosen. Of our reference candidates, β-actin, L13, and Tub are structure related. HPRT, PBGD, GAPDH, G6PDH, and phospholipase A2 (PLA) are metabolism related. TBP and RNA polymerase II (RPII) are transcription related, whereas albumin (Alb), β2M, and PPIA cannot be clearly put into above categories. The sequences of primers of these reference genes were listed in Supplementary Table 3. The most stable reference genes in exosome RNA were determined using Bestkeeper which was applied in order to visualize the centrifuged pellet. PLA ranked the most suitable reference gene in bile exosome RNA.

RNA sequencing data analyses

After sequencing, the raw reads should be filtered according to criterias: ribosomal RNA sequences, low quality reads and reads with sequence adaptors were removed. After the filtration, the clean reads were aligned to the human genome by Tophat-2 software. Both the value of “read-mismatches” and “read-gap-length” were set as 2, and the coverage as well as depth of the sequencing data were assessed. The resulting data were assembled into transcripts by the Cufflinks. The assembled transcripts were filtered using NCBI, RNA central, and GENCODE to select the known lncRNA and the known protein-coding mRNA. The relative expression levels of lncRNAs and mRNAs were measured as expected number of reads per kilobase of transcript sequence per million base pairs sequenced (RPKM) by HTSeq. DEGseq software was used to identify differentially expressed genes based on the following criteria: $|\log2 \text{ (fold change)}| > 1$ and $p < 0.05$. Target genes were predicted according to databases such as ncbi, RNAcentral, genecode and lncRNAdb. Pathway analysis (based on Kyoto encyclopedia of genes and genomes, KEGG, http://www.genome.jp) and gene ontology (GO) analysis were performed on the target genes of differentially expressed lncRNAs to predict the biological roles of them, and $p < 0.05$ used as threshold for defining significantly enriched GO terms and pathways. The co-expression network was constructed based on Pearson's correlation coefficient and z-score to explore the potential biological functions of lncRNAs.

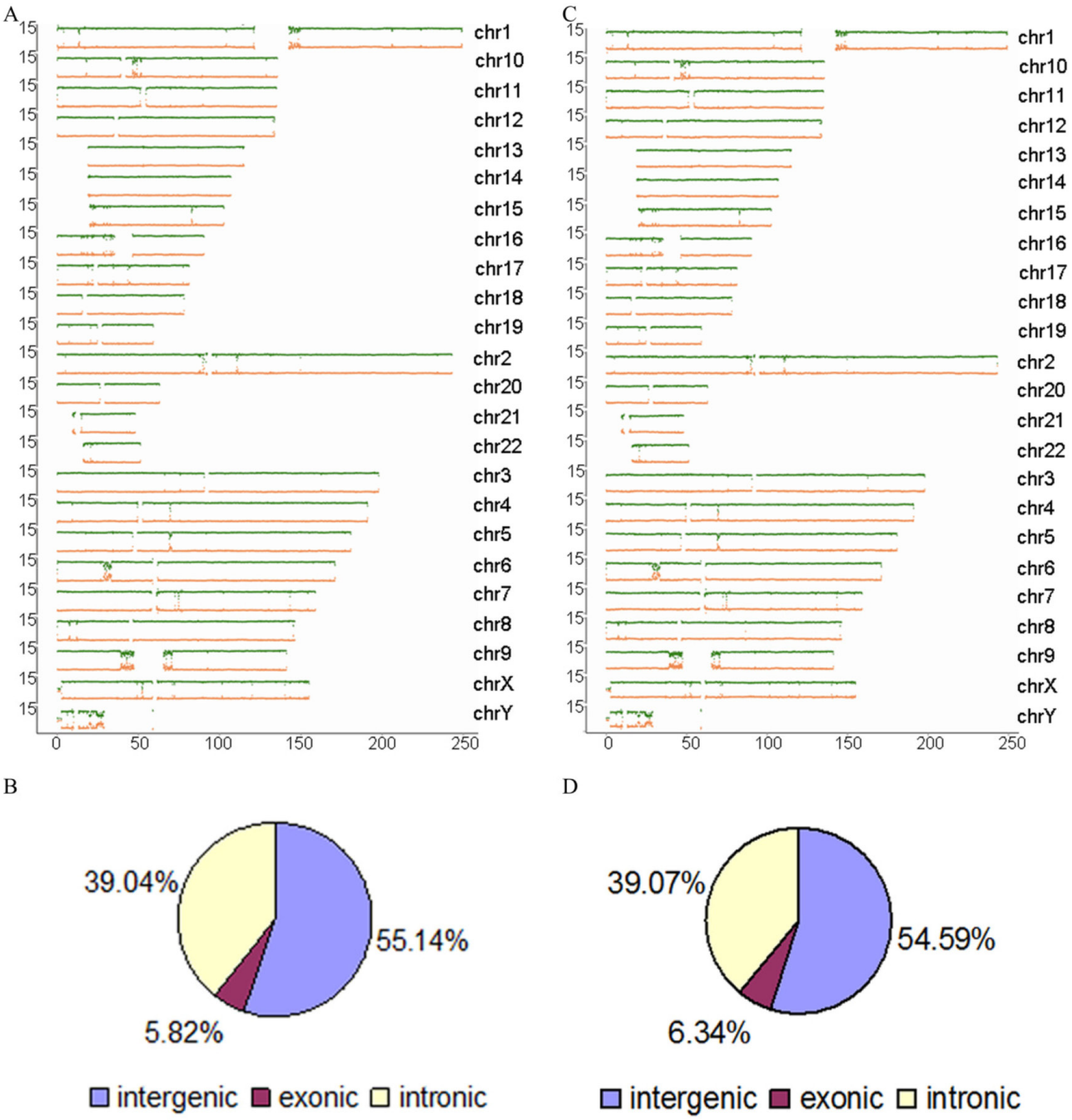

Supplementary Figure 1: Mapping of sequencing reads, and differentially expressed lncRNAs. **(A** and **C)** Overall coverage of the detected reads on human chromosome. "Chr" in (A) and (C) refers to chromosome. **(B** and **D)** General distribution of reads in different DNA regions.

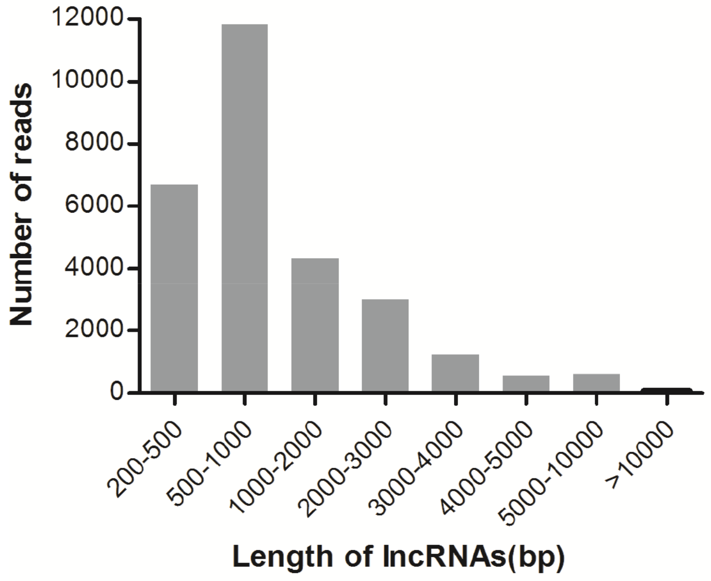

Supplementary Figure 2: Length distribution of exosomal lncRNAs.

Supplementary Table 1: Clinical and pathological characteristics of patients with CCA

	CCA (n = 35)	Control (n = 56)	*p*
Age, mean ± SE	66.3 ± 1.54	67.3 ± 1.63	0.664
Sex, male, n (%)	18 (51.4%)	26 (46.4%)	0.753
Tumer type			
iCCA, n (%)	4 (11.4%)		
dCCA, n (%)	22 (62.9%)		
pCCA, n (%)	9 (25.7%)		
TNM stage, n (%)			
I-II	16 (45.7%)		
III-IV	19 (54.3%)		

Intrahepatic cholangiocarcinomas (iCCAs) are located within the hepatic parenchyma; distal CCAs (dCCAs) are lacated from cystic duct to duodenal papilla; perihilar CCAs (pCCAs) are located between the two.

Supplementary Table 2: The list of lncRNA primers exosomal RNA level comparison between 3 CCAs and 3 control Primers

Genes	Genbank accession number	Primer sequence
ENST00000571590.1	Forward: 5' CGAGGTGGGAGGTTT 3'<br>Reverse: 5' TTCAGACGCTTATGACTTG 3'	ENSG00000242973.2
ENST00000518675.1	Forward: 5' CGAGGTGGGAGGTTT 3'<br>Reverse: 5' TTCAGACGCTTATGACTTG 3'	ENSG00000254211.1
ENST00000521904.1	Forward: 5' GCTGCGGGAGAACATA 3'<br>Reverse: 5' CAGCAGGAGGAAGTCAGTC 3'	ENSG00000253434.1
ENST00000531710.1	Forward: 5' CAGCAGACGAAGCCAGGAC 3'<br>Reverse: 5' GAGCGAGACTCCATCTTAGGG 3'	ENSG00000255348.1
ENST00000441146.1	Forward: 5' CTAGGAACAGTGCCTCCAAAAT3'<br>Reverse: 5' AACAGAGCTGACTCTACCGCT3'	ENSG00000229331.1
ENST00000554988.1	Forward: 5' ATCAACCCGCTCCAAGGAAT 3'<br>Reverse: 5' TCCCAGAACACATAGCGACA 3'	ENSG00000259001.2
ENST00000584779.1	Forward: 5' TTCACCACGTTAGCCAGGAT 3'<br>Reverse: 5' CAGGTGATCCTCCCACTTTG 3'	ENSG00000265073.1
ENST00000579049.1	Forward: 5' GGTGAGTGAGGCAGGAGAAT 3'<br>Reverse: 5' CCGGAGTCTCGCTCTGTC 3'	ENSG00000265094.1
ENST00000601661.1	Forward: 5' AAGCCAGGAGTTTGAGACCA 3'<br>Reverse: 5' CACCACAGCCTCCCAAGTAG 3'	ENSG00000269799.1
NR_003051	Forward: 5' GCTTCCCACTCCAAAGTCC 3'<br>Reverse: 5' CTAGAGGGAGCTGACGGATG 3'	RMRP
ENST00000450669.2	Forward: 5' CTGCTCGCTACACGACGTAA 3'<br>Reverse: 5' GCACATCAGGGTGAAATCCT 3'	ENSG00000230967.2
ENST00000588480.1	Forward: 5' TGCAGGAGGCAGTGAGACTA 3'<br>Reverse: 5' ACACACTCACAGGGCCAGAT 3'	ENSG00000267283.1
ENST00000607393.1	Forward: 5' TAGCTTCCGAGGGACTTCTC 3'<br>Reverse: 5' GGGCCGACTTAAACTCTGGT 3'	ENSG00000272043.1
ENST00000517758.1	Forward: 5' TAAATGGATTGGGACCCTCA 3'<br>Reverse: 5' CCTCCATCAACACGTTCACA 3'	ENSG00000253584.1
ENST00000607140.1	Forward: 5' GACTTCACCCGGCCTTTAAC 3'<br>Reverse: 5' TCAGAGGTGGCTTCATTGAT 3'	ENSG00000272524.1
NR_002794	Forward: 5' AGTGTGAGCCTGGTTTCACC 3'<br>Reverse: 5' CACCAGTATCGGCCTGAGTC 3'	TREML5P
ENST00000544663.1	Forward: 5' CCCACCTTCAGCAAACTCAT 3'<br>Reverse: 5' TTCTCCTCATTGGACCTGAGA 3'	ENSG00000256281.1
ENST00000454253.1	Forward: 5' GGAGCAACCTTCATCCAATG 3'<br>Reverse: 5' GCTCCCACGAGGAGAATAGG 3'	ENSG00000236641.1

Supplementary Table 3: The list of primers of reference mRNA

Genes	Primer sequence	Genbank accession number
HPRT	Forward: 5'-TGAGGATTTGGAAAGGGTGT -3'<br>Reverse:5'-GAGCACACAGAGGGCTACAA-3'	NM_000194
β-actin	Forward: 5'-TCCTCTCCCAAGTCCACACA -3'<br>Reverse:5'-GCACGAAGGCTCATCATTCA-3'	NM_001101.3
L13	Forward: 5'-CGGACCGTGCGAGGTAT -3'<br>Reverse:5'- CACCATCCGCTTTTTCTTGTC-3'	NM_000977.3
Tub	Forward: 5'-TGGAACCCACAGTCATTGATGA -3'<br>Reverse:5'- TGATCTCCTTGCCAATGGTGTA-3'	NM_003320.4
PBGD	Forward: 5'-GGCTGCAACGGCGGAA -3'<br>Reverse:5'-CCTGTGGTGGACATAGCAATGATT-3'	NM_000190.3
G6PDH	Forward: 5'-ATCGACCACTACCTGGGCAA -3'<br>Reverse:5'-TTCTGCATCACGTCCCGGA -3'	NM_001282587.1
PLA	Forward: 5'-AAGTTCTTGATCCCCAATGCTT-3'<br>Reverse:5'-GTCTGATAGGATGTGTTGGTTGC-3'	NM_000928.2
TBP	Forward: 5'-TTCGGAGAGTTCTGGGATTGTA-3'<br>Reverse:5'-TGGACTGTTCTTCACTCTTGGC-3'	NM_003194.4
RPII	Forward: 5'-GCACCACGTCCAATGACAT-3'<br>Reverse:5'-GTGCGGCTGCTTCCATAA-3'	NM_000937.4
Alb	Forward: 5'-TGCCCTGTGCAGAAGACTATCTA-3'<br>Reverse:5'-CGAGCTCAACAAGTGCAGTT-3'	NM_000477.5
β2M	Forward: 5'-AGCGTACTCCAAAGATTCAGGTT-3'<br>Reverse:5'-ATGATGCTGCTTACATGTCTCGAT-3'	NM_004048.2
PPIA	Forward: 5'-CATCTGCACTGCCAAGACTGAG-3'<br>Reverse:5'-TGCAATCCAGCTAGGCATG-3'	NM_021130.4
GAPDH	Forward: 5'-CCGGGAAACTGTGGCGTGATGG-3'<br>Reverse: 5'-AGGTGGAGGAGTGGGTGTCGCTGTT-3'	NM_001289746.1
Lusiferase	Forward: 5'-TTACACCCGAGGGGGATGAT-3'<br>Reverse: 5'-GTGTTCGTCTTCGTCCCAGT-3'	LC094340.1
